# Fenugreek Prevents the Development of STZ-Induced Diabetic Nephropathy in a Rat Model of Diabetes

**DOI:** 10.1155/2014/259368

**Published:** 2014-05-08

**Authors:** Yingli Jin, Yan Shi, Yinggang Zou, Chunsheng Miao, Bo Sun, Cai Li

**Affiliations:** ^1^Department of Pharmacology, College of Basic Medical Science, Jilin University, Xinmin Street 126, Changchun 130021, China; ^2^Department of Experimental Pharmacology and Toxicology, School of Pharmacy, Jilin University, Fujin Road 1266, Changchun 130021, China; ^3^The Second Hospital of Jilin University, Ziqiang Street 218, Changchun 130041, China

## Abstract

The present study aims to examine the protective effect of fenugreek and the underlying mechanism against the development of diabetic nephropathy (DN) in streptozotocin- (STZ-) induced diabetic rats. A rat model of diabetes was successfully established by direct injection of STZ and then the rats were administered an interventional treatment of fenugreek. Parameters of renal function, including blood glucose, albuminuria, hemoglobin A1c (HbA1c), dimethyl formamide (DMF), blood urine nitrogen (BUN), serum creatinine (Scr), and kidney index (KI), were detected in the three groups (Con, DN, and DF). Oxidative stress was determined by the activity of antioxidase. Extracellular matrix (ECM) accumulation and other morphological alterations were evaluated by means of immunohistochemistry and electron microscope. Quantitive (q)PCR was employed to detect the mRNA expression of transforming growth factor-*β*1 (TGF-*β*1) and connective tissue growth factor (CTGF) and protein expression was determined with western blot analysis. DN rats in the present study demonstrated a significant renal dysfunction, ECM accumulation, pathological alteration, and oxidative stress, while the symptoms were evidently reduced by fenugreek treatment. Furthermore, the upregulation of TGF-*β*1 and CTGF at a transcriptional and translational level in DN rats was distinctly inhibited by fenugreek. Consequently, fenugreek prevents DN development in a STZ-induced diabetic rat model.

## 1. Introduction 

Diabetes mellitus has been becoming a global health problem. Diabetes is a life threatening disorder since it strongly increases the risk of cardiovascular complications, such as coronary artery disease, myocardial infarction, hypertension, and dyslipidemia [1–3]. The cardiovascular injury mainly target two organs, eye and kidney [4], which makes diabetic nephropathy (DN) become one of the most common complications of diabetic patients. DN is the leading cause of end-stage renal disease (ESRD) which is responsible for disabilities and high mortality rates in patients with diabetes. However, the mechanism underlying DN development is largely unclear. Therefore, studies should be persistently conducted for a better understanding of DN pathogenic mechanisms and novel therapeutic agents.

DN is histologically characterized by an accumulation of extracellular matrix (ECM) in the glomerulus and the interstitium [5]. Although the mechanisms underlying DN development have not been completely elucidated, the ECM accumulation and the following fibrosis in diabetic kidney have been considered as crucial in the renal dysfunction and morphological changes of glomerulus. Oxidative stress and several growth factors such as transforming growth factor-*β*1 (TGF-*β*1) and connective growth factor (CTGF) have been recognized to involve in the pathogenic mechanisms.

In diabetic patients and animal models, ECM accumulation and renal dysfunction are always accompanied with severe oxidative stress in the diabetic kidney. Furthermore, treatments with antioxidants such as vitamin E, taurine or lipoic acid reduce the DN-related pathological alterations in the glomeruli and albuminuria in diabetic rats [6–8]. These findings demonstrate the implication of oxidative stress in ECM accumulation and renal dysfunction. Afterwards, many scholars investigated the potential mechanisms. They found that the oxidative stress induced by hyperglycemia aggravated the ECM accumulation of diabetic kidney through the upregulation of TGF-*β*1 [9], as the increased TGF-*β*1 could stimulate the ECM production and suppress its degradation. TGF-*β*1 has been identified as the crucial mediator of ECM accumulation in the diabetic kidney [10]. Another important mediator is CTGF which delivers signals received from TGF-*β*1 to induce ECM synthesis and renal fibrosis in the DN development [11, 12]. Therefore, seeking for an agent that could alleviate renal oxidative stress and block TGF-*β*1/CTGF signaling pathway may be a good choice for DN therapy.

Fenugreek (*Trigonella foenum-graecum*) is used as the condiment, a supplement to wheat and maize flour for bread making, and as the constituent of the daily diet of the general population in many countries. Currently, a number of studies on animal and human have indicated that extractions from fenugreek seed have antidiabetic, hypocholesterolemic, and antioxidant effect. Therefore, fenugreek is a perfect choice for DN therapy. Fenugreek pharmacological features have been demonstrated in diabetes mellitus research by detecting its functions on peripheral glucose utilization, insulin secretagogue actions, and the effect on the gum fiber in the intestines [13–18]. In the present study, we aim to examine the potential protective effect of fenugreek on kidney in the streptozotocin- (STZ-) induced diabetic rats and further to confirm whether fenugreek could reduce DN risk through alleviating renal oxidative stress and inhibiting TGF-*β*1/CTGF signaling pathway in glomeruli of the STZ-induced diabetic rats.

## 2. Materials and Methods

### 2.1. Drug

Fenugreek seed used in the present study is native to Sichuan Province of China and was purchased from Changchun Medicine Herbs Supply and Sales Company (Changchun, China). The fenugreek seed has been identified by Professor Guangshu Wang who is expert in the Chinese herbal medicine research. Active ingredients of the fenugreek seed have been isolated and identified by the team of Professor Zhongying Liu in Department of Pharmacology of Jilin University. The yield of the active ingredients was approximate 0.74% and it predominantly consisted of polysaccharides (about 89.0%). Molecular weight between 150 and 180 kD of the polysaccharides is up to 70.6% (mainly galactomannan), <150 kD 18.4%, and others 11%. In the present study, fenugreek seeds were ground to powder for the following experiments. Fenugreek seed powder (200 g) was put into 2000 mL distilled water and boiled for 0.5 hour to obtain the decoction. Then the decoction was cooled for 1 h at room temperature and filtered twice through a coarse sieve. Finally, the filtrate was concentrated by rotavapour (Buchi, R-210, Germany) at 50°C into a thick paste [19].

### 2.2. Animals

Sprague-Dawley rats (half male and half female, 10 weeks old, weighing 200–250 g) were obtained from the Experimental Animal Center of Jilin University. They were housed in a pathogen-free facility with free access to the standard dried chow diet and water throughout the period of study. All the animal experiments were conducted following internationally recognized guidelines on animal welfare, as well as the principles issued by the State Scientific and Technological Commission (Beijing, China).

### 2.3. Induction of Diabetes and Experimental Design

Following acclimatization for 7 days, SD rats were administered fast for 12 h and then received intraperitoneal injection with 50 mg/kg streptozotocin (STZ, Sigma, St. Louis, MO, USA) in citrate solution (0.1 M citric acid and 0.2 M sodium phosphate, pH 4.5). Following this (after 3 days), blood glucose levels was detected utilizing One-Touch strip. Rats with blood glucose concentrations above 13.9 mmol/L were considered as diabetic and were used following experiments [5, 20]. A week later, diabetic rats were randomly administered fenugreek (9 g seed powder/kg, DF group, *n* = 10) daily for 12 weeks or treated with vehicle control (DN group, *n* = 10), respectively. Normal rats as control (Con group, *n* = 10) were administered citrate buffer alone and treated with vehicle control for 12 weeks. There was an equal distribution of male to female rats in the 3 groups following randomization.

### 2.4. Measurement of Blood Glucose and Renal Function

The rats were sacrificed at 12 weeks after STZ injection and their kidney tissues were dissected and weighed. The kidney index (KI) was calculated as 1000 × kidney weight/body weight. Blood glucose, hemoglobin A1c (HbA1c), dimethyl formamide (DMF), and 24 h urinary albumin excretion (albuminuria) were determined by kit, respectively, from Huili Company, China. All kits were used in accordance with the manufacturer's instructions. Blood urine nitrogen (BUN) and serum creatinine (Scr) concentrations were measured utilizing a Hitachi 7150 Biochemical Autoanalyzer (Hitachi, Tokyo, Japan).

### 2.5. Determination of Glomerular Volume

At least six different sections were randomly selected from each group of rat kidney for histomorphometric studies. The area (*μ*m^2^) of a minimum of 30 glomerular sections from each rat was detected under light microscopy by means of the MIAS-2000 image analysis system (Zhisheng). The glomerular volume (*V*) was calculated according to the formula *V* = *β*/*k* × [*A*]^3/2^, *β* = 1.38, *k* = 1.10 [21].

### 2.6. Antioxidation Assays

The Cu/Zn superoxide dismutase (SOD) activity was assessed following the protocol as previously described [22]. Briefly, sodium citrate buffer (pH 9.4, 50 mmol/L) (2.6 mL) consisted of 30 mmol/L Xanthine (dissolved in 1 mol/L NaOH), 0.8 mmol/L XTT (3-bis(4-methoxy-6-nitro)-benzenesulfonic acid hydrate), 3 mmol/L EDTA dissolved in buffer at 50°C, and 0.05 mL of sample solution containing SOD or water. At last, 500 mU/mL Xanthine oxidase solution 0.02 mL was added into the reaction and the absorbance value was monitored at 470 nm (25°C) for 30 s.

Catalase (CAT) activity was detected according to Aebi's description [23]. The homogenate was prepared with 50 mmol/L phosphate buffer, pH 7.0, a drop of Triton X 100 and centrifuged at 15,000 g for 15 min at 4°C. 3.0 mL of phosphate buffer, 0.05 mL of 90 mmol/L hydrogen peroxide solution, and 0.02 mL of extract or water were added. The absorbance was read at 240 nm for 30 s.

Glutathione peroxidase (GSH-PX) activity was determined by the method described in the study of Paglia and Valentine [24]. The reaction mixture contained phosphate buffer 2.6 mL (pH 7.0, 100 mmol/L) with 3 mmol/L EDTA, 0.05 mL GSH solution (10 mg/mL), 0.1 mL of 10 mg/mL GR, 0.05 mL of 10 mg/mL NADPH-Na salt, 0.1 mL of hydrogen peroxide solution (90 mmol/L), and 0.1 mL of sample. The GSH-PX activity was determined by the reduced absorbance because of the consumption of NADPH, and the absorbance change is at 340 nm.

Renal malondialdehyde (MDA) content was assessed following the literature [25]. This method bases on the test of thiobarbituric acid (TBA) reaction. Renal samples were weighed and homogenized (1/10 w/v) in ice-cold 1.15% KCl solution and mixed with 0.1 mL of 8.1% sodium dodecyl sulfate (SDS), 0.75 mL of 20% acetic acid, and 0.75 mL of 0.8% TBA solution. The mixture was made up to 2.0 mL with distilled water and heated at 95°C for 60 min. Following cooling with tap water, 0.5 mL of distilled water and 2.5 mL of n-butanol/pyridine mixture (15 : 1 v/v) were added and shaken vigorously. The mixture was centrifuged at 4000 g for 10 min and the absorbance of the organic layer (upper layer) was measured at 532 nm. The TBA reaction was standardized by the analysis of tetraethoxypropane (TEP) standard solutions, which yield MDA, mole for mole, under the described reaction conditions.

### 2.7. Quantitive (q)PCR

Total RNA was extracted from renal cortical tissues utilizing TRIzol reagent (Gibco, Beijing, China) and reversely transcribed into cDNA using SuperScript II reverse transcriptase at 37°C for 1 h. PCR amplification was performed in duplicate at 94°C for 2 min and subjected to 30 cycles of 94°C for 30 s, 55°C for 30 s, and 72°C for 30 s, followed by extension at 72°C for 7 min using specific primers and Taq DNA Polymerase (DingGuo Biotech, Beijing, China). The sequences of primers are listed as follows: TGF-*β*1 (forward: 5′-CCA AGG AGA CGG AAT ACA GG-3′, reverse: 5′-GTG TTG GTT GTA GAG GGC AAG-3′), CTGF (forward: 5′-CTAAGACCTGTGGAATGGGC-3′, reverse: 5′-CTCAAAGATGTCATTGCCCCC-3′), and *β*-actin (forward: 5′-CAT CTC TTG CTC GAA GTC CA-3′, reverse: 5′-ATC ATG TTT GAG ACC TTC AAC A-3′). The PCR products were separated by agarose gel electrophoresis and visualized by ethidium bromide staining. The relative levels of target gene mRNA transcripts to control GAPDH were analyzed by densimetric scanning using a Tanon-1000 Gel Image System (Shanghai, China), as our previous studies [26].

### 2.8. Western Blot Assays

The renal cortical tissues of rats in different groups were respectively homogenized in lysis buffer and centrifuged. The supernatants were collected. Following quantification of protein concentrations, the tissue lysates (40 *μ*g protein/lane) were separated by SDS-PAGE on a 7.5% polyacrylamide gel, followed by transferring onto nitrocellulose membranes (GE Healthcare, Beijing, China). Subsequently, the membranes were blocked in 5% skim dry milk in PBS and incubated with polyclonal rabbit anti-rat CTGF (1 : 200 dilution, Wuhan Boster Biological Technology, Ltd., Wuhan, China), anti-rat TGF-*β*1 (1 : 100 dilution, Wuhan Boster Biological Technology, Ltd., Wuhan, China), or anti-*β*-actin (1 : 1000 dilution, Wuhan Boster Biological Technology, Ltd., Wuhan, China) at 4°C overnight. The bound antibodies were detected with horseradish peroxidase- (HRP-) conjugated anti-rabbit IgG and visualized using an enhanced chemiluminescence kit, according to the manufacturers' instruction (GE Healthcare, Beijing, China). The relative levels of target proteins to control *β*-actin were determined by densimetric scanning.

### 2.9. Histological Examinations

One portion of kidney tissues was fixed in 10% buffered formalin and embedded in paraffin for a light microscopic study. The kidney tissue sections (4 *μ*m) were stained with periodic acid-Schiff reagent (PAS) and examined under a light microscope (Olympus PM-10AO, Japan). The degrees of mesangial matrix expansion in different groups of rats were determined as PAS-positive staining in the mesangial region excluding cellular elements. The percentages of PAS-positive areas in 10 glomeruli selected randomly from two sections of each rat (10 rats per group) were analyzed utilizing Leica Q500MC image analysis software with blinded manner, as described in our previous study [26].

A portion of renal cortex tissues was cut into small pieces, prefixed in 2.5% glutaraldehyde (0.2 mol/L cacodylate buffer, pH 7.4) for 4 h, postfixed in 1% buffered sodium tetroxide for 1 h, and embedded in Epon. The ultrathin renal cortex sections were examined with a JEM-1200 EX electron microscope.

### 2.10. Immunohistochemistry

The protein levels of fibronectin (FN), type IV collagen (Col IV), CTGF, and TGF-*β*1 in renal tissue sections were examined by immunochemistry, as described in our previous study [26]. Briefly, the renal tissue sections were treated with polyclonal rabbit anti-rat fibronectin, Col IV (Santa Cruz Biotechnology, Santa Cruz, CA), and anti-rat CTGF and TGF-*β*1 (Boster Biological Technology Wuhan, China). The bound antibodies were detected with HRP-anti-rabbit IgG and diaminobenzidine (DAB), followed by counterstaining with hematoxylin. Negative controls were incubated with PBS. The percentages of positive staining areas in the glomerulus were determined semiquantitatively using computer imaging analysis system.

### 2.11. Statistical Analyses

Data are presented as mean ± SD. The difference among the three groups was analyzed by one-way ANOVA and post hoc analysis by Bonferroni correction. The difference between two groups was analyzed by Student's *t*-test using SSPS software. Sex difference was also accounted for in the statistical analysis. A *P* value of <0.05 was considered statistically significant.

## 3. Results

### 3.1. Fenugreek Reduced Blood Glucose Levels and Improved Renal Functions of Diabetic Rats

In the present study, diabetic rat model with blood glucose level ≥13.9 mmol/L was successfully established by intraperitoneal injection of STZ. The mean ± SD of blood glucose concentration in the DN group was 18.09 ± 4.1 mmol/L, nearly three times of that in the Con group. By contrast, the elevated blood glucose level of diabetic rats in DF group was evidently decreased following fenugreek treatment (*P* < 0.05), which demonstrated that fenugreek could reduce blood glucose levels of the diabetic rats (Table 1). To examine the renal injury and dysfunction induced by STZ injection, and whether fenugreek could improve renal function of diabetic rats or not, indicators of renal function were detected, including albuminuria, HbA1c, DMF, BUN, Scr, and KI. As a result, each of the indicators was significantly elevated in the DN group compared with the Con group (*P* < 0.05, resp.). These data further indicated that the STZ-induced DN rat model had been successfully established, and the diabetic rats had suffered from renal dysfunction. While, when fenugreek treatment was performed in the DF group, each of the elevated parameters decreased strikingly to a normal range compared with the Con group (*P* > 0.05, resp., Table 1). Therefore, these findings demonstrated that the STZ-induced diabetic rats were subjected to renal injury and dysfunction and that fenugreek exerts a protective effect on kidneys of the diabetic rats.

### 3.2. Fenugreek Inhibited ECM Accumulation in Glomeruli of Diabetic Rats

**Figure 1:**
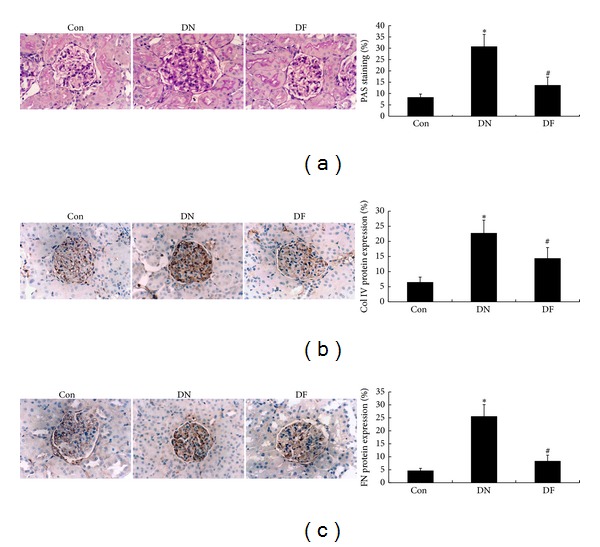
Reduced ECM accumulation in the diabetic kidney after fenugreek treatment. ECM accumulation in the kidney was measured by PAS staining (a) under a light microscope (×400). Significant increase of PAS-positive staining in the mesangial region, obvious proliferation of mesangial cell, and capillaries collapse were found in the STZ-induced diabetic group (DN), but they did not appear in normal nondiabetic group (Con), and were observed little in fenugreek-treated diabetic group (DF) with no significant difference compared with Con group. Quantitative analysis for the percentage of PAS in the glomerulus by the computer image analysis system is presented as the histogram. In addition, protein levels of Col IV (b) and FN (c) as the two main components of ECM were detected by immunohistochemistry. They had a significant increase in DN group compared with Con group, respectively, and decreased obviously after fenugreek treatment in DF group. Results of the quantitative analysis are also expressed by the histograms in (b) and (c), respectively. *P < 0.05 versus Con; ^#^ *P* < 0.05 versus DN.

ECM accumulation is one of the characteristic pathological alterations in glomeruli of DN patients and animal models. Collagen and FN are the predominant components of ECM and generally considered as the predictors of ECM accumulation. In the present study, glomerular ECM accumulation in the three groups was evaluated by means of the histological examination and the expression of Col IV and FN was detected utilizing immunohistochemistry. Compared with the Con group, the DN group exhibited more PAS-positive materials in the renal cortex of diabetic rats (*P* < 0.05, Figure 1(a)). Meanwhile, the results of immunohistochemistry further revealed that the expression of Col IV (Figure 1(b)) and FN (Figure 1(c)) was distinctly upregulated in the DN group compared with the control group. However, in DF group, the pathological changes were significantly reduced by fenugreek treatment. These results indicated that fenugreek could inhibit the ECM accumulation in glomeruli of diabetic rats.

### 3.3. Fenugreek Alleviated Glomerular Morphological Alterations of Diabetic Rats

**Figure 2:**
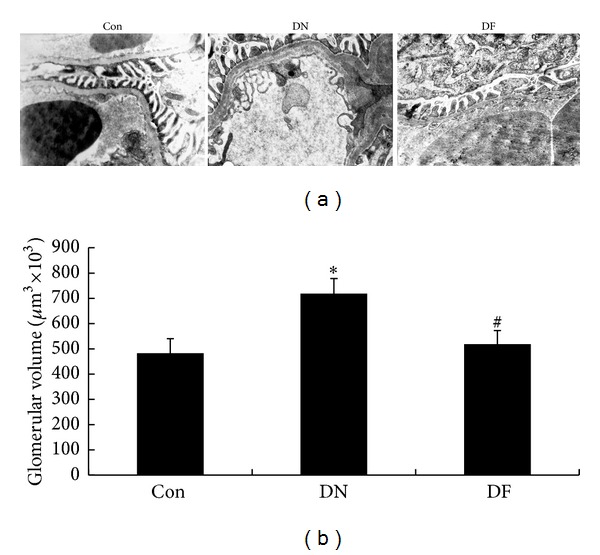
Alleviated glomerular morphological changes in the diabetic kidney after fenugreek treatment. Except for the morphological changes observed in Figure 1, other changes such as segmental thickening of glomerular basement membranes, widely fused foot processes podocytes, and excessively deposited mesangial matrix were also found in DN group under electron microscope (×20 000) and disappeared after fenugreek treatment in DF group (a). Besides, glomerular volume of the diabetic kidney in DN group enlarged significantly compared with the Con group and then diminished after fenugreek treatment in DF group (b). *P < 0.05 versus Con; ^#^ *P* < 0.05 versus DN.

Except for ECM accumulation, other morphological changes in glomeruli were assessed under electron microscopy. Three typical alterations were detected in the glomeruli of diabetic rats in DN group, including segmental thickening of glomerular basement membranes, widely fused foot processes podocytes, and excessively deposited mesangial matrix, while these ultrastructural abnormalities were evidently prevented by fenugreek in DF group (Figure 2(a)). Therefore, the findings demonstrated that fenugreek could attenuate morphological alterations by inhibiting the ECM accumulation in glomeruli of diabetic rats. In our further investigations, glomerular volume was measured under electron microscopy and quantified to be as the measure index of glomerular hypertrophy. As a result, glomerular volume was significantly enlarged in DN group versus Con group (*P* < 0.05) and distinctly reduced by fenugreek treatment in DF group versus DN group (*P* < 0.05, Figure 2(b)). This suggested that fenugreek could evidently mitigate glomerular hypertrophy of diabetic rats.

### 3.4. Fenugreek Relieved Glomerular Oxidative Stress and Lipid Peroxidation of Diabetic Rats

**Figure 3:**
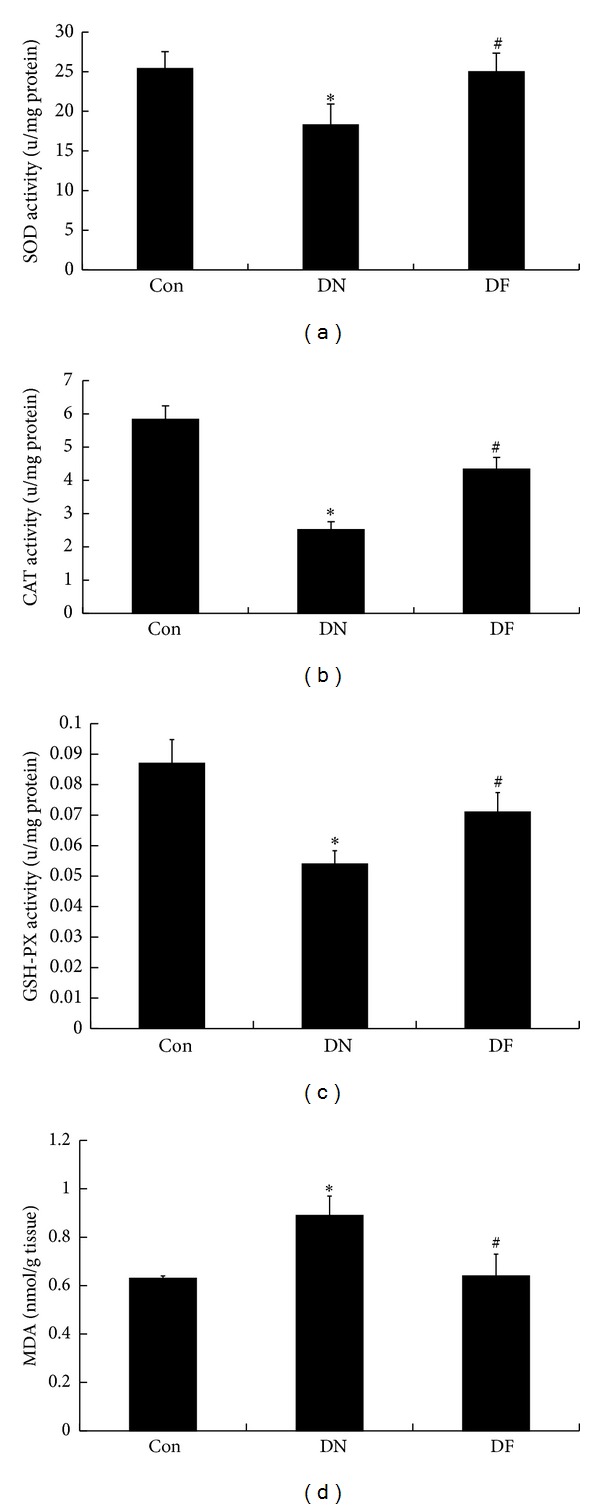
Increased activity of SOD, CAT, and GSH-PX and decreased MDA level after fenugreek treatment. The alleviated oxidative stress and lipid peroxidation in diabetic kidney were relieved by the fenugreek treatment through promoting the activity of SOD (a), CAT (b), and GSH-PX (c). Meanwhile, the increased MDA level of diabetic kidney was successfully reduced by the fenugreek treatment (d). *P < 0.05 versus Con; ^#^ *P* < 0.05 versus DN.

To elucidate the implication of oxidative stress and lipid peroxidation in the progression of renal dysfunction and morphological alterations, as well as the potential effect of fenugreek, glomerular SOD, CAT, and GSH-PX activity of rats in the three groups were detected, and the MDA level which is the end-product of lipid peroxidation was also assessed. Compared with the Con group, the DN group demonstrated a significant decrease in the activity of glomerular SOD (*P* < 0.05, Figure 3(a)), CAT (*P* < 0.05, Figure 3(b)), and GSH-PX (*P* < 0.05, Figure 3(c)), together with an evident increase in MDA level (*P* < 0.05, Figure 3(d)). These findings suggested that oxidative stress and lipid peroxidation were involved in the DN development. When the comparison was performed between DN and DF groups, it was identified that glomerular SOD, CAT, and GSH-PX of DF group were significantly activated in the DF group (*P* < 0.05, resp.), accompanied with the evidently dropped MDA level (*P* < 0.05). The results suggested that fenugreek had an antioxidant activity and could relieve the glomerular oxidative stress and lipid peroxidation in the STZ-induced diabetic rats.

### 3.5. Fenugreek Inhibited the Expression of mRNA and Protein of TGF-*β*1 in Glomeruli of Diabetic Rats

**Figure 4:**
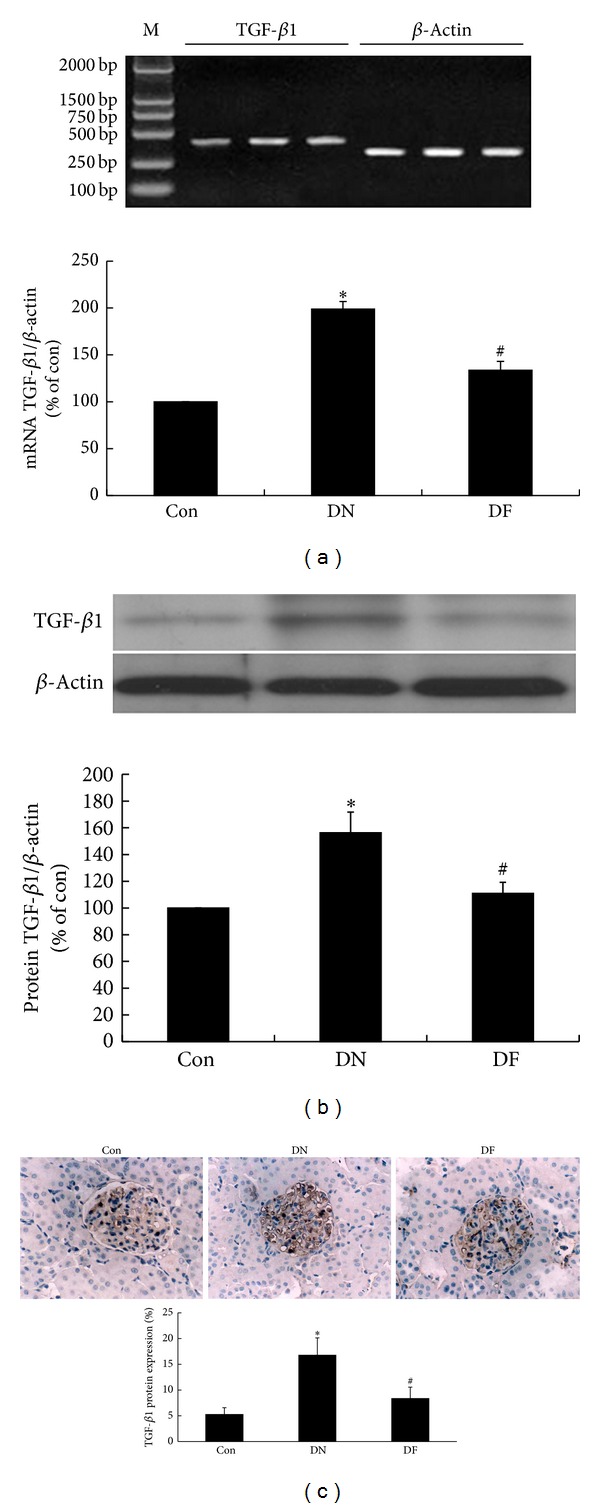
Fenugreek suppressed the mRNA and protein expressions of TGF-*β*1 in diabetic kidney. MRNA and protein levels of TGF-*β*1 (a and b) were tested by qPCR and western blot assay, respectively. Beta-actin was used as the internal standard in each sample. Data for relative quantity of TGF-*β*1 mRNA and protein were conducted by densitometric analysis. At the same time, histopathological examinations were performed by immunohistochemistry (c). They suggested the same results. *P < 0.05 versus Con; ^#^ *P* < 0.05 versus DN.

Accumulating evidence suggests that TGF-*β*1 participates in DN development by stimulating ECM production and suppressing its degradation. TGF-*β*1 has been recognized as a potential target for DN therapy. To evaluate the effect of fenugreek on TGF-*β*1, the expression of mRNA and protein of TGF-*β*1 in glomeruli was determined by qPCR and western blot analysis, respectively. Consequently, both mRNA (Figure 4(a)) and protein (Figure 4(b)) levels of TGF-*β*1 were significantly upregulated in the DN group compared with the Con group; then the increased expression was strikingly reduced by fenugreek in the DF group. This indicated that fenugreek could inhibit the expression of mRNA and protein of TGF-*β*1 in glomeruli of diabetic rats. Subsequently, the conjecture was further confirmed by the results of immunohistochemistry test in the present study; that is, the protein expression of TGF-*β*1 (Figure 4(c)) was evidently upregulated in the renal cortex of diabetic rats in the DN group compared with the Con group but was distinctly inhibited by fenugreek in the DF group compared with the DN group.

### 3.6. Fenugreek Inhibited the Expression of mRNA and Protein of CTGF in Glomeruli of Diabetic Rats

**Figure 5:**
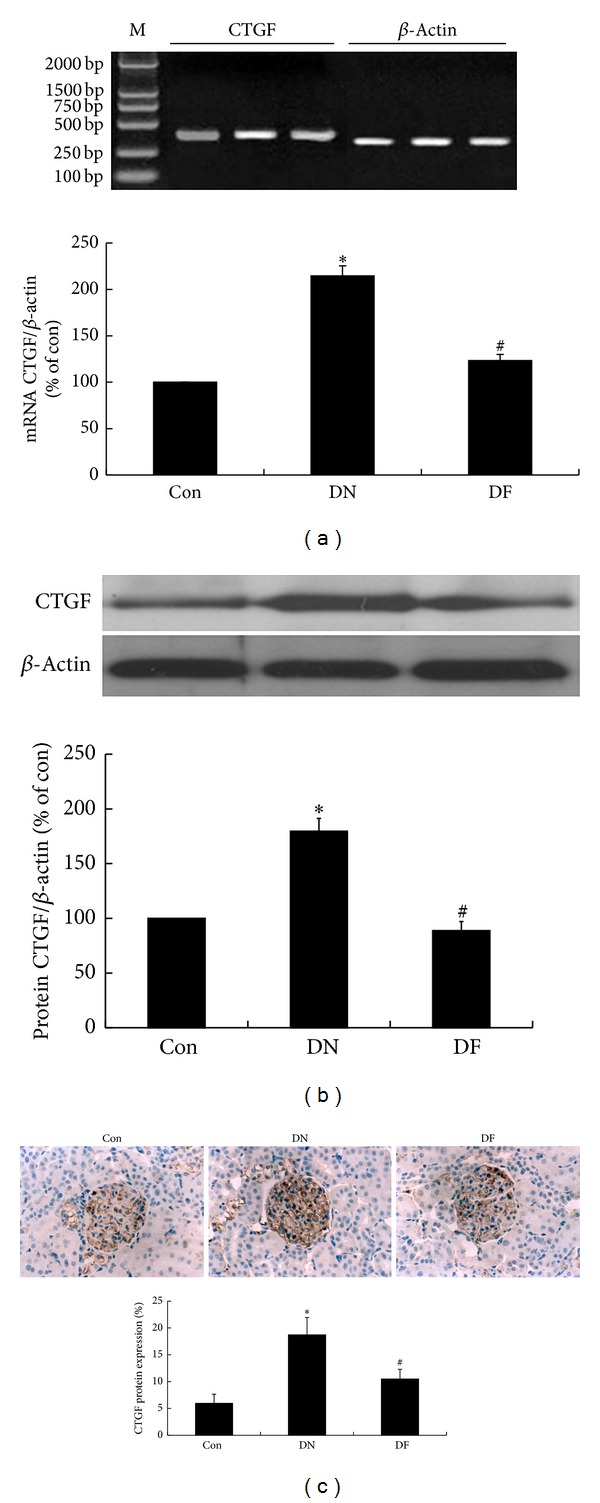
Fenugreek inhibited mRNA and protein expressions of CTGF. Tests and calculations for mRNA and protein levels of CTGF were same with TGF-*β*1 in Figure 4. Upregulated mRNA and protein expressions of CTGF were found in diabetic kidney of DN group compared with Con group; then they were downregulated by fenugreek treatment in DF group (a and b). At last, the results were further confirmed by the immunohistochemistry test (c). *P < 0.05 versus Con; ^#^ *P* < 0.05 versus DN.

TGF-*β*1 signaling pathway has been confirmed as a vital pathway to involve in DN development, and CTGF as one of the crucial downstream mediators also has been report to participate in the process. CTGF may be a second important potential target for DN therapy. Whether fenugreek has an effect on CTGF, too? QPCR was employed to detect the the mRNA expression of CTGF and protein expression was determined with western blot analysis. As a result, the mRNA (Figure 5(a)) and protein (Figure 5(b)) level of CTGF had a coordinated alteration with TGF-*β*1 in the three groups. Therefore, the results confirmed the involvement of CTGF in DN development, and demonstrated that fenugreek could inhibit the mRNA and protein expression of CTGF in glomeruli of diabetic rats. In a similar manner, the inhibitory effect of fenugreek on the mRNA and protein expression of CTGF were further confirmed by the results of immunohistochemistry test in the present study, that is, CTGF (Figure 5(c)) had a significant increase in protein expression in the renal cortex of diabetic rats in the DN group compared with the Con group, and had a evident decrease following fenugreek treatment in the DF group compared with the DN group.

## 4. Discussion

As one of the major complications of diabetes mellitus, DN becomes a leading cause of end-stage renal failure worldwide. Typical morphological alterations of DN kidney are represented by glomerular basement membrane (GBM) thickening, mesangial expansion, broadening and effacement of podocyte foot processes, glomerular hypertrophy, glomerulosclerosis, and tubulointerstitial fibrosis [27–29]. These alterations predominantly attribute to the ECM accumulation in the glomeruli and the interstitium of diabetic kidney [30]. Based on the previous studies, oxidative stress induced by hyperglycemia was considered as the promoter of DN development in the present study. Signals induced by oxidative stress were delivered through TGF-*β*1/CTGF pathway to activate ECM accumulation and the following morphological alterations and finally cause the renal dysfunction and DN development in diabetic rats. Fenugreek was selected as the potential drug against DN with its antioxidation and nontoxicity. To investigate the protective effect of fenugreek against DN development and provide evidence for the hypothesis that fenugreek may reduce DN risk through alleviating renal oxidative stress and inhibiting TGF-*β*1/CTGF signaling pathway, we conducted the present study and preliminarily confirmed the hypothesis.

The STZ-induced diabetic rat model has been widely used in DN research. In the present study, we successfully established the rat model of diabetes according to the previous description [5, 20] (Table 1). Generally, DN is functionally characterized by the raised urinary protein and renal dysfunction. In the present study, the DN group exhibited significant increase in albuminuria, HbA1c, DMF, BUN, Scr, and KI compared with the Con group (Table 1). This demonstrated that the STZ-induced diabetic rats had been subjected to renal dysfunction. Renal dysfunction is usually caused by the alterations of structural/cellular basis in DN kidney, including ECM accumulation, GBM thickening, mesangial cell matrix deposition, and the effacement of podocyte foot processes [4, 31]. In the present study, the morphological alterations referred to above were demonstrated in the DN group. Most importantly, they were evidently attenuated by fenugreek treatment (Figures 1 and 2), accompanied with the improvement of renal function (Table 1). Therefore, it is concluded that the renal dysfunction is associated with the renal morphological alterations, and fenugreek could reduce the risk of renal dysfunction by preventing injuries to the structural/cellular basis.

As we all know, the structural/cellular basis is easily destroyed by the oxidative stress which has been verified to participate in the pathogenesis of diabetic complications, including DN [32, 33]. Oxidative stress refers to the imbalance between the antioxidative defense system and the oxidative system [34, 35]. Oxidative stress is initially induced by the hyperglycemic condition and predisposition in the diabetic kidney [36], and then the oxidative stress stimulates the excessive production of reactive oxygen species that are toxic to the cell, particularly the cell membrane in which these radicals interact with the lipid bilayer and produce lipid peroxides. Endogenous antioxidant enzymes including SOD, CAT, and GSH-PX are responsible for the detoxification of the deleterious oxygen species [37]. In the present study, the activity of SOD, CAT, and GSH-PX in glomeruli of diabetic rats was evidently inhibited along with a distinct increase in MDA level. By contrast, following fenugreek treatment, the activity of all the enzymes displayed a significant increase accompanied with the obvious decrease in MDA level (Figure 3). These results demonstrate that fenugreek possesses antioxidant activity and could improve the antioxidative defense system of the DN kidney.

Although many factors are implicated in the development and progression of DN, the ECM accumulation has been recognized as a crucial determiner in DN development. Proteins, collagen, and FN are the predominant components of the ECM. In the present study, significant upregulation of Col IV and FN was detected in the DN glomeruli of diabetic rats. TGF-*β*1 has been identified as the regulator of ECM accumulation. In diabetic patients and animal models, progressively raised expression of mRNA and protein of TGF-*β*1 was detected and always accompanied with the increased production of ECM components, whereas blockade of the overexpression of TGF-*β*1 could prevent the pathological alterations, such as ECM accumulation and GBM thickening [38–41]. In the present study, the diabetic rats had an upregulation of TGF-*β*1 in mRNA and protein expression (Figure 4), which was consistent with the results of previous studies. The findings confirm the implication of TGF-*β*1 in the development of DN. Furthermore, the increased mRNA and protein levels of TGF-*β*1 along with the excessive oxidative stress in DN glomeruli provide an evidence for the conjecture that oxidative stress may induce ECM accumulation through upregulating TGF-*β*1 expression. When fenugreek treatment was performed on the diabetic rats, the upregulated mRNA and protein expression of TGF-*β*1 in glomeruli decreased strikingly, accompanied with the relieved oxidative stress and ECM accumulation. These further demonstrate that fenugreek may reduce the morphological alterations of DN kidney through its antioxidant activity and TGF-*β*1 pathway.

CTGF, a 349-amino acid cysteine-rich peptide, is another important growth factor involved in DN development. CTGF has been recognized as the downstream mediator of TGF-*β*1 in regulating matrix metabolism during the fibrotic process [42–44]. A number of studies on diabetic human and animal models indicate that mRNA and protein expression of TGF-*β*1 and CTGF have a coordinated increase in the diabetic glomeruli; meanwhile, the activated signaling of matrix metabolism by TGF-*β*1 is markedly affected by CTGF. These suggest the involvement of TGF-*β*1 and CTGF in DN development, as well as the association between TGF-*β*1 and CTGF in matrix metabolism. In the present study, the same results were obtained; that is, mRNA and protein expression of CTGF had a significant increase in DN glomeruli of diabetic rats (Figure 5), along with the increased mRNA and protein expression of TGF-*β*1, as well as the upregulated Col IV and FN. Following fenugreek treatment, the increased mRNA and protein levels of CTGF decreased strikingly; meanwhile, the increased mRNA and protein levels of TGF-*β*1 together with the upregulated Col IV and FN obviously decreased as well. These results demonstrate that CTGF participates in ECM accumulation in glomeruli of diabetic rats, and fenugreek may reduce the ECM accumulation by inhibiting TGF-*β*1/CTGF signaling pathway.

## 5. Conclusion

In the present study, DN development was demonstrated in the STZ-induced diabetic rats, such as ECM accumulation, renal dysfunction, and the excessive oxidant stress along with the over expression of TGF-*β*1 and CTGF at transcriptional and translational level. Following fenugreek treatment, DN symptoms were significantly eliminated, which was represented by the reduced blood glucose, improved renal functions, suppressed ECM accumulation, and other morphological alterations, as well as the coordinately relieved oxidant stress together with the restrained TGF-*β*1/CTGF signaling pathway. Findings in the present study confirm the protective effect of fenugreek against the development and progression of DN and preliminarily demonstrate the hypothesis that fenugreek may reduce DN risk through alleviating renal oxidative stress and suppressing TGF-*β*1/CTGF signaling pathway. However, many further studies should be conducted to find more detailed evidence for the hypothesis. Nevertheless, fenugreek has a protective effect on the diabetic kidney and prevents the DN development in diabetic rats induced by STZ injection. Fenugreek may be a promising drug for DN therapy.

## Figures and Tables

**Table 1:** Decreased blood glucose and alleviated renal dysfunctions after fenugreek treatment.

	Con	DN	DF
Glucose (mmol/L)	6.12 ± 1.56	18.09 ± 4.1*	7.42 ± 2.68^#^
Albuminuria (mg/day)	7.28 ± 3.24	17.23 ± 3.26*	10.13 ± 3.03^#^
HbAlc (%)	6.39 ± 1.52	12.95 ± 4.36*	8.12 ± 1.38^#^
DMF (mmol/L)	1.82 ± 0.39	2.93 ± 0.68*	2.05 ± 0.41^#^
BUN (mmol/L)	6.59 ± 1.43	17.82 ± 3.01*	9.14 ± 1.59^#^
Scr (*μ*mol/L)	25.90 ± 2.83	51.91 ± 5.69*	25.34 ± 2.61^#^
KI (g/g)	2.72 ± 0.61	5.94 ± 0.51*	4.69 ± 0.41^#^

Twelve weeks after fenugreek treatment, renal function parameters of the nondiabetic control group (Con), STZ-induced diabetic group (DN), and the fenugreek-treated diabetic group (DF) were tested. Data are presented as mean ± SD from 10 animals (*n* = 10) for each group. The kidney index (KI) was calculated as 1000 × kidney weight/body weight. *P < 0.05 versus Con; ^#^ *P* < 0.05 versus DN.
